# Navigating formula shortages: associations of parental perspectives on transitioning to alternative infant formulas for cow's milk protein allergy during the 2022 national formula shortage

**DOI:** 10.3389/falgy.2023.1333570

**Published:** 2024-01-08

**Authors:** Abigail L. Fabbrini, Andrew A. Farrar, Jerry M. Brown, Lea V. Oliveros, Jared Florio, Jesse Beacker, Luke Lamos, Jessica V. Baran, Michael J. Wilsey

**Affiliations:** ^1^ Office of Medical Education, Kansas City University College of Osteopathic Medicine, Kansas City, MO, United States; ^2^ Office of Medical Education, Florida Atlantic University Charles E. Schmidt College of Medicine, Boca Raton, FL, United States; ^3^ Office of Medical Education, Alabama College of Osteopathic Medicine, Dothan, AL, United States; ^4^ Department of Pediatrics, University of South Florida Morsani College of Medicine, Tampa, FL, United States

**Keywords:** COVID-19 pandemic, extensively hydrolyzed formula, amino acid formula, formula shortage crisis, AAF, eHF

## Abstract

The COVID-19 pandemic led to supply chain disruptions causing a severe shortage of infant formula. The shortage impacted parents of infants with cow's milk protein allergy (CMPA) who rely on specialized formulas. However, research on parent perspectives during formula shortages is limited. We aimed to understand the factors guiding parents' decisions when transitioning to alternative amino acid formula (AAF) or extensively hydrolyzed formula (eHF) during the national formula shortage. We conducted a survey using the ZSMoments platform and found that before the shortage, parents valued safety (83%), tolerability (78%), and reputability (78%) as primary factors in selecting eHFs and AAFs. Post-shortage, formula tolerability (86%), assurance (84%), and safety (80%) gained more importance. Among those switching eHF (*n* = 54), health care provider recommendations (81%), reputability (78%), taste (78%), and tolerability (78%) were rated as “extremely important.” Among those switching AAF (*n* = 26), top factors included tolerability (77%), assurance (73%), safety (73%), cost-effectiveness (73%), and formula trustworthiness (73%). These data suggest that parents carefully weigh various factors when managing their child's CMPA and transitioning to different AAF or eHF options.

## 1 Introduction

The COVID-19 pandemic disrupted supply chains world-wide, impacting many products including infant formula (1). In February 2022, a major manufacturing facility recall exacerbated formula shortages in the United States (1), which impacted parents of infants with cow's milk protein allergy (CMPA) requiring specialized formulas, such as extensively hydrolyzed (eHF) and amino acid (AAF) formulas (2, 3). Healthcare providers, notably pediatricians, faced the challenge of assisting parents, particularly those requiring hypoallergenic formula (4).

CMPA is common in infancy, with an estimated prevalence of 2%–7.5% in the first year of life (3). Infants with CMPA can experience a wide range of symptoms, including urticaria, colic, vomiting, and diarrhea (3, 5, 6). CMPA arises from an abnormal immune response to cow's milk proteins. Symptoms can vary widely in severity and presentation among infants affected (5, 6). Immediate IgE-mediated reactions occur shortly after milk protein consumption, leading to hives, vomiting, diarrhea and numerous other symptoms (3, 7). Delayed non-IgE-mediated reactions manifest hours to days later with gastrointestinal problems, poor weight gain, irritability and numerous other symptoms (2, 8).

Management can include eliminating cow's milk protein from breastfeeding mothers' diets and starting hypoallergenic formulas for formula-fed infants (3). However, the national formula shortage forced many parents to switch to different hypoallergenic options (1). In 2020, the United States underwent an infant formula shortage stemming from COVID-19-related import restrictions (1). The predicament intensified in 2021 amid a global supply chain disruption and reached a peak in February 2022, attributed to a substantial recall from a company supplying 40% of U.S. infant formula (1, 4). Grocery stores, pharmacies, and online retailers all felt the impact of this scarcity (1, 3, 9). The nationwide shortage of infant formula carried significant risks for formula-dependent infants, leaving parents uncertain about ensuring safe feeding options.

Hence, parents faced the task of evaluating multiple aspects when contemplating switches between hypoallergenic formulas, yet there is limited research on how parents perceive formula shortages. Exploring parent perspectives is crucial for obtaining practical insights into challenges such as finding alternatives and managing switches due to formula availability concerns. This observational study delved into the factors parents considered when transitioning between Extensively Hydrolyzed Formulas (eHFs) and Amino Acid-Based Formulas (AAFs) amid the national formula shortage. We hypothesized that parents of infants with Cow's Milk Protein Allergy (CMPA), who had to switch formulas during the shortage, would prioritize formula tolerability, assurance, and safety as the most critical factors in their decision-making process.

## 2 Methods

This cross-sectional study investigates parent experiences switching between eHFs or AAFs during the infant formula shortage between January 2022 and November 2022. For the purposes of this study, Nutramigen (Mead Johnson Nutrition, Evansville, IN), Alimentum (Abbott Nutrition, Columbus, OH), and Extensive HA (Gerber Products Co. Arlington, VA) will be referred to as EHF-1, EHF-2, and EHF-3, respectively. Puramino (Mead Johnson Nutrition, Evansville, IN), Alfamino (Nestlé Infant Nutrition Inc., Florham Park, NJ), Elecare (Abbott Nutrition, Columbus, OH), and Neocate (Nutricia, Liverpool, UK) will be referred to as AAF-1, AAF-2, AAF-3, and AAF-4, respectively.

Inclusion criteria included parents of infants and toddlers (≤24 months) needing eHF or AAF formula to relieve symptoms of CMPA and parents who switched their infant's formula from eHF-2/3 to eHF-1, and who switched from AAF-2/3/4 to AAF-1 during the formula shortage period. Eligible parents completed an online survey through the ZSMoments, a mobile application that allows for the rapid, secure documentation of real-time patient data (5, 6). This study received exempt status from the Institutional Review Board (IRB), indicating that it met the criteria for exemption from full IRB review.

Survey questions were developed based on a review of the literature and discussions with healthcare professionals who manage infants with CMPA. The survey examined driving factors such as safety, efficacy, and tolerability and the impact on parents who were forced to choose alternative eHFs and AAFs. The questionnaire consisted of yes-or-no questions and questions rated on a scale from 1 to 10. We considered ratings of 8–10 as “extremely important.” The survey was administered in English and took approximately 30 min to complete.

Data from the surveys were analyzed using SPSS analytical software (IBM Corporation, Armonk, NY, USA). Descriptive statistics were used to summarize the data and chi-square tests were conducted to determine the association between demographic variables and formula switch details. Statistical significance was set at *p* < 0.05.

## 3 Results

### 3.1 Overall

**Figure 1:**
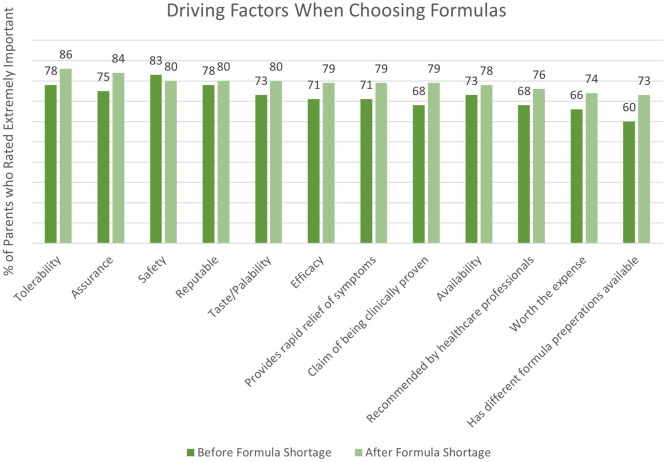
Driving factors for parents when choosing a hypoallergenic formula before and after the formula shortage.

**Figure 2:**
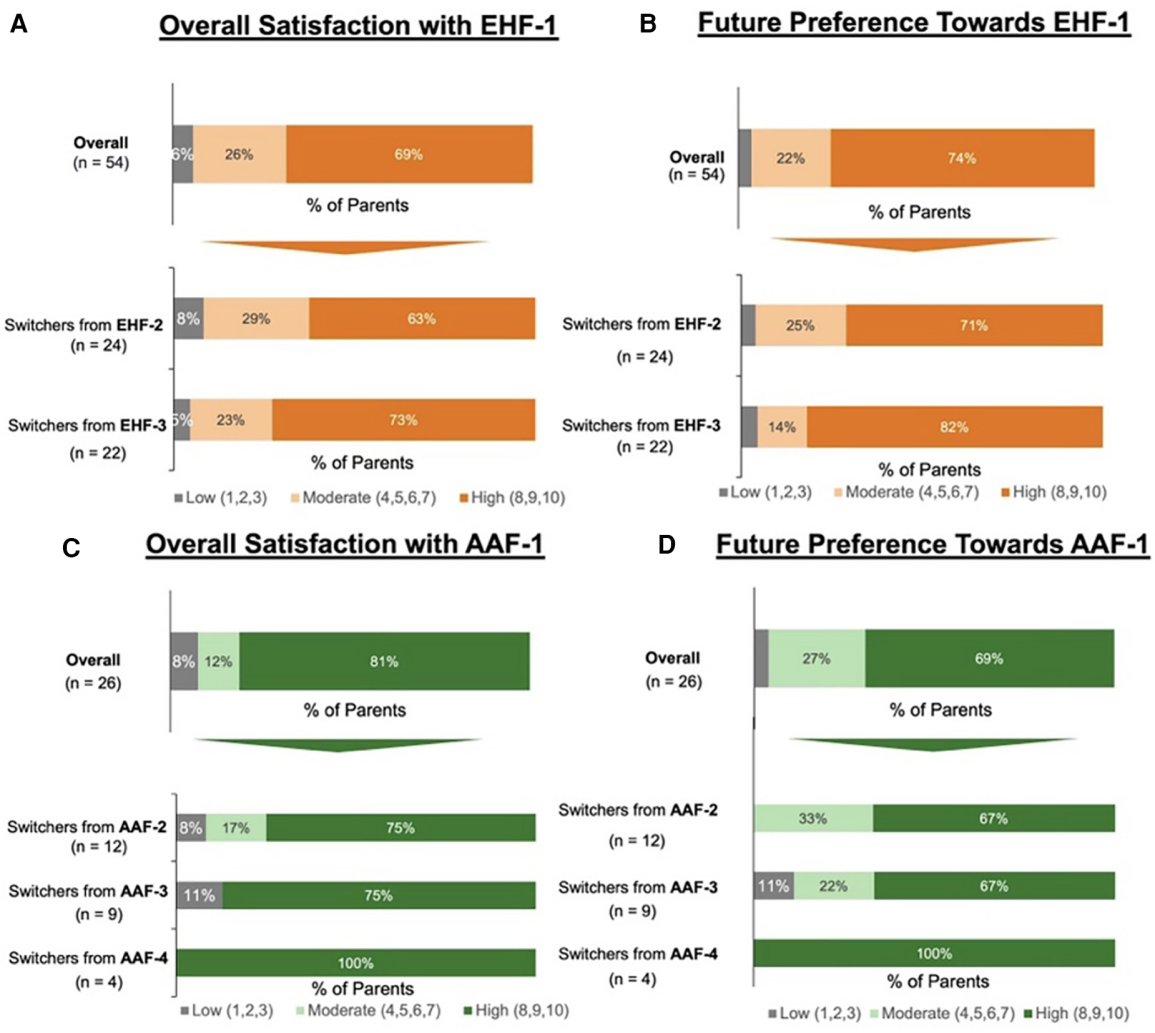
(**A**) Parents reported levle of satisfaction with eHF-1 following the switch; (**B**) percent of parents who reported they would continue using eHF-1 in the future. (**C**) Parents reported level of satisfaction with AAF-1 following the switch; (**D**) percent of parents who reported they would continue using AAF-1 in the future.

A combined total of 80 parents were surveyed on the factors they considered most important when switching their infants’ formula. Figure 1 depicts driving factors for parents when choosing an eHF or AAF before and after the formula shortage. The survey results revealed that safety (83%), tolerability (78%), and formula trustworthiness (78%) were the most frequently reported important factors that parents prioritized when selecting an eHF or AAF before the shortage. Conversely, formula tolerability (86%), assurance (84%), and safety (80%) gained prominence after the shortage. Parents' opinions on the new formula, as well as future formula preference, is depicted in Figure 2.

### 3.2 EHF switch

Of the 80 parents surveyed, 54 parents switched their infant's formula to EHF-1. Of these 54 parents, 28 (52%) had infants less than 12 months of age and 26 (48%) had infants 13–24 months of age. Of those that switched to EHF-1, 24 (44%) switched from EHF-2, 22 (41%) switched from EHF-3, and 8 (15%) switched from other formulas. When switching from one eHF to another (*n* = 54) after the formula shortage, the factors most frequently rated as “extremely important” for parents were the formula being recommended by healthcare providers (81%), formula trustworthiness (78%), taste/palatability (78%), and tolerability (78%). Figure 2 depicts the overall satisfaction and future preference of eHF-1 after switching formulas. Of the 54 parents, 69% were highly satisfied and 74% reported future preference for eHF-1.

### 3.3 AAF switch

Of the 80 parents surveyed, 26 parents switched the infant's formula from one AAF to another. Of these 26 parents, 11 (42%) had infants less than 12 months of age and 15 (58%) had infants 13–24 months of age. Of those that switched to AAF-1, 12 (46%) switched from AAF-2, 9 (35%) switched from AAF-3, 4 (15%) switched from AAF-4, and 1 (4%) switched from other formulas. When switching from one AAF to another (*n* = 26), the factors most frequently rated as “extremely important” for parents were tolerability (77%), assurance (73%), safety (73%), worth the expense (73%), and brand trustworthiness (73%). Figure 2 depicts the overall satisfaction and future preference of AAF-1 after switching formulas. Of the 26 parents, 81% were highly satisfied and 69% reported future preference for eHF-1.

## 4 Discussion

In this study, we conducted a survey among parents of infants managing CMPA with eHFs or AAFs. The aim was to understand the factors shaping their choices when selecting alternative formulas amidst the 2022 infant formula shortage. We report a cohort of 80 parents, 54 who switched their infant's formula between eHFs and 26 who switched their infant's formula between AAFs.

Our survey data indicated that parents prioritize formula tolerability, assurance, and safety as pivotal factors when switching formulas. These factors highlight influences on their decision-making process and underscore their concerns for their infant's well-being, emphasizing the importance of healthcare guidance and dependable formula options. Notably, health care providers' recommendation, formula trustworthiness, taste, and tolerability were rated “extremely important” when selecting an eHF. For switching to an AAF, the key factors were tolerability, assurance, safety, cost-effectiveness, and formula trustworthiness.

Recommended CMPA management in formula-fed infants involves using hypoallergenic formulas, such as extensively hydrolyzed formulas (eHF) or amino acid formulas (AAF) (3, 8). These formulas are designed to be less allergenic, with small broken down proteins or free amino acids, reducing reactions in infants with CMPA. Some infants might not find relief from symptoms with eHFs and may need AAFs. In our study, infants were receiving eHF or AAF based on healthcare provider recommendations before switching (2). Hypoallergenic formulas allow CMPA infants to receive necessary nutrition while avoiding allergenic proteins (2, 3, 5, 6, 8).

Research on parent perspectives during formula shortages is limited, yet vital for practical insights into formula availability challenges, including finding alternatives and navigating switches. Collaborative efforts between healthcare providers and parents can mitigate formula shortage challenges, ensuring infants' nutrition with minimal stress. In August 2022, a study found about 35% of US consumers sought infant formula during the crisis (9). Common resources included online searches, multiple stores, and enlisting help from friends and family. Health agency recommendations, like brand switching and breastfeeding, were used less frequently (9). Potentially unsafe infant feeding practices like dilution, sharing breastmilk, and homemade formulas emerged during shortages (10). Useful resources for parents included WIC, healthcare providers, other parents, friends, and lactation consultants (10).

There are several limitations to this study. Firstly, the sample size of our cohort may not be representative of the larger population of parents affected by the infant formula shortage. Secondly, reliance on self-reported data from parents could introduce recall bias. The survey focused solely on parents who had already switched to eHF or AAF, potentially introducing selection bias. Despite these potential limitations, our study provides valuable insights into the factors influencing parental decision-making during the infant formula shortage.

## 5 Conclusion

This study sheds light on the pivotal factors influencing parental decisions during formula shortages. Our results underscore the significance of healthcare providers emphasizing formula tolerance, safety, and assurance for parents when discussing formula options. These findings offer insights that can potentially inform clinical practice and future research. However, larger studies are warranted to validate these preliminary results.

## Data Availability

The raw data supporting the conclusions of this article will be made available by the authors, without undue reservation.
