# Corrigendum: Navigating formula shortages: associations of parental perspectives on transitioning to alternative infant formulas for cow’s milk protein allergy during the 2022 national formula shortage

**DOI:** 10.3389/falgy.2024.1380490

**Published:** 2024-02-28

**Authors:** Abigail L. Fabbrini, Andrew A. Farrar, Jerry M. Brown, Lea V. Oliveros, Jared Florio, Jesse Beacker, Luke Lamos, Jessica V. Baran, Michael J. Wilsey

**Affiliations:** ^1^ Office of Medical Education, Kansas City University College of Osteopathic Medicine, Kansas City, MO, United States; ^2^ Office of Medical Education, Florida Atlantic University Charles E. Schmidt College of Medicine, Boca Raton, FL, United States; ^3^ Office of Medical Education, Alabama College of Osteopathic Medicine, Dothan, AL, United States; ^4^ Department of Pediatrics, University of South Florida Morsani College of Medicine, Tampa, FL, United States

**Keywords:** COVID-19 pandemic, extensively hydrolyzed formula, amino acid formula, formula shortage crisis, AAF, eHF

A Corrigendum on Navigating formula shortages: associations of parental perspectives on transitioning to alternative infant formulas for cow’s milk protein allergy during the 2022 national formula shortage By Fabbrini AL, Farrar AA, Brown JM, Oliveros LV, Florio J, Beacker J, Lamos L, Baran JV and Wilsey MJ (2024). Front. Allergy. 4:1333570. doi: 10.3389/falgy.2023.1333570

**Text Correction**

In the published article, there was an error. An incorrect **Ethics statement** was inserted. This statement previously stated:

“The studies involving humans were approved by Johns Hopkins All Children's Hospital Institutional Review Board. The studies were conducted in accordance with the local legislation and institutional requirements. The participants provided their written informed consent to participate in this study.”

The corrected statement appears below:

“This study underwent review by the Advarra Institutional Review Board (IRB) and was determined to be exempt from ongoing IRB oversight as per the US Department of Health and Human Services regulations outlined in 45 CFR 46.104(d)(2). This determination reflects the IRB's assessment that the research meets the ethical standards and criteria for exemption.”

The authors apologize for this error and state that this does not change the scientific conclusions of the article in any way. The original article has been updated.

