# Supplementary material for: Navigating formula shortages: associations of parental perspectives on transitioning to alternative infant formulas for cow's milk protein allergy during the 2022 national formula shortage
Source: Front Allergy. 2024 Jan 8;4:1333570. doi: 10.3389/falgy.2023.1333570 (PMC10801258; doi:10.3389/falgy.2023.1333570)
Supplement: Supplementary file 1 [file Table1.docx]

Parent Survey:

1. Are you or anyone in your family affiliated in any way with any of the following on a full time or part time basis: the FDA, a pharmaceutical or biotech manufacturing company; a market research, public relations, advertising firm, or healthcare consulting firm?
2. Which state are you residing in currently?
3. What is the current age of your child (in months)?
4. Please indicate the gender of the child under your care.
5. Please think about the child under your care. What conditions/symptoms has the child experienced? Select all that apply.
   1. Cow’s Milk Protein Allergy (CMPA)
   2. Other food allergy (excluding CMPA)
   3. Skin allergies (e.g. eczema, atopic dermatitis)
   4. Blood in stool
   5. Colic
   6. Frequent crying
   7. Gassiness
   8. Fussiness
   9. Constipation
   10. Reflux/spit-ups
   11. Diarrhea
   12. Vomiting
   13. Others
6. How long ago did your child first experience each of the following symptoms (in months)?
   1. Cow’s Milk Protein Allergy (CMPA)
   2. Other food allergy (excluding CMPA)
   3. Skin allergies (e.g. eczema, atopic dermatitis)
   4. Blood in stool
   5. Colic
   6. Frequent crying
   7. Gassiness
   8. Fussiness
   9. Constipation
   10. Reflux/spit-ups
   11. Diarrhea
   12. Vomiting
   13. Others
7. Did you use a specialty formula to relieve the child of these conditions/symptoms?
   1. Yes
   2. No
8. Did you need to switch between infant formula products for your child in the past 10 months due to supply shortages/recall of products?
   1. Yes, I had to switch products primarily due to shortage issues.
   2. Yes, I had to switch products primarily due to recall of products.
   3. Others (Please Specify ____)
   4. No, I have not switched infant formula products due to supply shortages/recall of products.
9. You previously indicated having switched infant formula products, which product do you use currently?
   1. Nutramigen
   2. Alimentum
   3. Gerber Extensive HA
   4. Other EH brands (Extensively Hydrolyzed Products, please specify ____)
   5. PurAmino
   6. Neocate
   7. Elecare
   8. Alfamino
   9. Other AA brands (Amino Acid Based Products, please specify ____)
   10. Other (Please specify ____)
10. You previously indicated having switched infant formula products, which product did you use before the shortage/recall crisis?
    1. Nutramigen
    2. Alimentum
    3. Gerber Extensive HA
    4. Other EH brands (Extensively Hydrolyzed Products, please specify ____)
    5. PurAmino
    6. Neocate
    7. Elecare
    8. Alfamino
    9. Other AA brands (Amino Acid Based Products, please specify ____)
    10. Other (Please specify ____)
11. **]** Consider each of the following specialty formula brands. For how long had you been using each of the following brands for your child **before the formula shortage/recall crisis started** (in months)?
    1. Nutramigen
    2. Alimentum
    3. Gerber Extensive HA
    4. PurAmino
    5. Neocate
    6. Elecare
    7. Alfamino
    8. Other (please specify ____)
12. Did you switch to Nutramigen or PurAmino?
13. Kindly elaborate your reasons for switching to that formula?
14. Considering the list of attributes mentioned below, please rate on a scale from 1-10, 1 being not at all important and 10 being extremely important, how important each attribute was when deciding on an infant formula brand for your baby BEFORE the formula shortage/recall crisis started.
    1. Worth the expense
    2. Safety
    3. Efficacy
    4. Provides rapid relief of symptoms
    5. Tolerability
    6. Taste/Palatability
    7. Availability
    8. Recommended by healthcare professionals
    9. Different formula preparations available
    10. Reputable
    11. Assurance
    12. Claim of being clinically proven
15. Considering the list of attributes mentioned below, please rate on a scale from 1-10, 1 being not at all important and 10 being extremely important, how important each attribute was when deciding on an infant formula brand for your baby AFTER the formula shortage/recall crisis started.
    1. Worth the expense
    2. Safety
    3. Efficacy
    4. Provides rapid relief of symptoms
    5. Tolerability
    6. Taste/Palatability
    7. Availability
    8. Recommended by healthcare professionals
    9. Different formula preparations available
    10. Reputable
    11. Assurance
    12. Claim of being clinically proven
16. Number of days it took my child to successfully drink/accept the product without difficulty
17. Based on your experience with the following infant formula brand/s, how would you rate, each brand on the attributes shown below? Please rate on a scale from 1 to 10, 1 being performs very poorly and 10 being performs extremely well. Please choose 0 if you have not used the product or do not have enough experience to rate your satisfaction.
    1. Nutramigen
    2. Alimentum
    3. Gerber Extensive HA
    4. PurAmino
    5. Neocate
    6. Elecare
    7. Alfamino
18. Based on your experience with the following infant formula brand/s, how would you rate your **overall** **level of satisfaction** with each brand? Please rate on a scale from 1 to 10, 1 being performs very poorly and 10 being performs extremely well. Please choose 0 if you have not used the product or do not have enough experience to rate your satisfaction.
    1. Nutramigen
    2. Alimentum
    3. Gerber Extensive HA
    4. PurAmino
    5. Neocate
    6. Elecare
    7. Alfamino
19. Please consider the below infant formula products. How likely are you to continue to use them in the future, even after the shortage/recall crisis has been resolved? Please rate on a scale from 1 to 10, 1 being not at all likely to use and 10 being highly likely to use.
    1. Nutramigen
    2. Alimentum
    3. Gerber Extensive HA
    4. PurAmino
    5. Neocate
    6. Elecare
    7. Alfamino
20. If you indicated that you are **likely to continue to use** the following products (rating a 7-10), please indicate **why you want to continue using** **the product, even after the shortage/recall crisis has been resolved**.
    1. Nutramigen
    2. PurAmino
21. If you indicated that you are **NOT likely to continue to use** following products (rating a 1-4), please indicate **why you do not want to continue using the product, after the shortage/recall crisis has been resolved**.
    1. Nutramigen
    2. PurAmino
22. Assuming that the formula shortage/crisis is over, how likely are you to go back to your previous brand? Please rate on a scale from 1 to 10, 1 being not at all likely to use and 10 being highly likely to use.
